# Effects of Biannual Azithromycin Mass Drug Administration on Malaria in Malawian Children: A Cluster-Randomized Trial

**DOI:** 10.4269/ajtmh.19-0619

**Published:** 2020-04-27

**Authors:** John D. Hart, Lyson Samikwa, Feston Sikina, Khumbo Kalua, Jeremy D. Keenan, Thomas M. Lietman, Sarah E. Burr, Robin L. Bailey

**Affiliations:** 1 London School of Hygiene and Tropical Medicine, London, United Kingdom;; 2 College of Medicine, University of Malawi, Blantyre, Malawi;; 3 Blantyre Institute for Community Outreach, Blantyre, Malawi;; 4 Francis I Proctor Foundation and Department of Ophthalmology, University of California, San Francisco, San Francisco, California

## Abstract

Reductions in malaria morbidity have been reported following azithromycin mass drug administration (MDA) for trachoma. The recent Macrolides Oraux pour Reduire les Deces avec un Oeil sur la Resistance (MORDOR) trial reported a reduction in child mortality following biannual azithromycin MDA. Here, we investigate the effects of azithromycin MDA on malaria at the MORDOR-Malawi study site. A cluster-randomized double-blind placebo-controlled trial, with 15 clusters per arm, was conducted. House-to-house census was updated biannually, and azithromycin or placebo syrup was distributed to children aged 1–59 months for a total of four biannual distributions. At baseline, 12-month, and 24-month follow-up visits, a random sample of 1,200 children was assessed for malaria with thick and thin blood smears and hemoglobin measurement. In the community-level analysis, there was no difference in the prevalence of parasitemia (1.0% lower in azithromycin-treated communities; 95% CI: −8.2 to 6.1), gametocytemia (0.7% lower in azithromycin-treated communities; 95% CI: −2.8 to 1.5), or anemia (1.7% lower in azithromycin-treated communities; 95% CI: −8.1 to 4.6) between placebo and azithromycin communities. Further interrogation of the data at the individual level, both per-protocol (including only those who received treatment 6 months previously) and by intention-to-treat, did not identify differences in parasitemia between treatment arms. In contrast to several previous reports, this study did not show an effect of azithromycin MDA on malaria parasitemia at the community or individual levels.

## INTRODUCTION

The Macrolides Oraux pour Reduire les Deces avec un Oeil sur la Resistance (MORDOR) trial, conducted in Niger, Malawi, and Tanzania, demonstrated a reduction in child mortality following biannual mass drug administration (MDA) with azithromycin.^1^ The mechanism through which such a reduction in child mortality may occur is not clear. Azithromycin is a broad spectrum antibiotic with a relatively long half-life which is used in the treatment of pneumonia and diarrhea but also displays antimalarial activity.^2,3^ Field trials of the effects of azithromycin MDA on malaria infection and symptoms have previously reported reductions in malariometric indices; and recent results from the Niger MORDOR site indicate an association between azithromycin MDA and lower parasitemia.^3–7^ It is feasible that the mortality benefit seen with azithromycin MDA may be due, at least in part, to a decrease in malaria prevalence or severity.

This study reports malaria parasitemia, parasite density, and gametocytemia data from the MORDOR Malawi study site, aiming to improve our understanding of the effects of azithromycin MDA on malaria infection. Samples were collected from children in villages representative of the MORDOR trial and used the same biannual census updates and cluster-randomized trial structure to make the results as representative as possible of the wider study area and to assess community-level effects of any outcomes. The hypothesis for this study was that azithromycin MDA would reduce the community prevalence of malaria compared with placebo.

## METHODS

### Trial design.

The randomization unit for the MORDOR trial in Malawi was defined as the catchment area of a health surveillance assistant (HSA), approximately 1,000 total population. Communities with a population < 200 or > 2,000 on a pre-baseline census were excluded. Thirty communities were randomly selected from the pool of communities for the MORDOR trial for follow-up as part of this malaria prevalence study. The randomization was stratified to produce six communities in each of the five geographical zones of Mangochi district for geographical generalizability and for logistical reasons regarding fieldwork. Biannual census updates were performed, and communities received study drug in the same treatment rounds as the MORDOR trial.

### Participants.

All children aged 1–59 months and weighing ≥ 3.8 kg were eligible for treatment at each of four biannual mass distributions. At the baseline, 12-month, and 24-month follow-up visits, guardians of a randomly selected sample of 40 children per community were asked to provide written informed consent for finger-prick blood samples. The procedures and study were explained by trained local nursing staff who subsequently collected thick and thin blood smears if consent was obtained. Illiterate guardians provided a thumb print to acknowledge consent.

### Interventions.

Azithromycin was administered at a dose of 20 mg/kg. Children old enough to stand received an approximate dose estimated from their height, and younger children were weighed. The placebo bottles and suspension appeared identical to azithromycin. Distribution of drug took place after sample collection was complete and was performed by the HSAs and field-workers conducting house-to-house visits. Guardians were asked to inform the HSA of any adverse events that occurred within 7 days of receiving study drug. Health surveillance assistants subsequently informed the study team.

### Outcomes.

The primary prespecified outcome was prevalence of malaria parasites on thick blood smears in children aged 1–59 months. Prespecified secondary outcomes included parasite density, gametocyte prevalence and density, hemoglobin concentration, and presence of anemia (Hb < 11 g/dL). Primary analyses were by intention-to-treat, and per-protocol analyses were secondary.

### Sample collection.

Sample collection took place during the baseline visit (May–July 2015) and at 12-month and 24-month visits (April–June 2016 and 2017, respectively), approximately 6 months after the second and fourth treatment rounds. Sample collection involved selected children receiving a finger stick and thick and thin blood smears collected on a single slide (hemoglobin measurement was performed using a Hemocue 201 device (Ängelholm, Sweden)). Slides were labeled with a random number and barcode and scanned using the data collection app to link to census data. The thin smear was fixed with absolute methanol and the slide stained with 8% Giemsa. Parasite density was assessed from the thick smear by two independent slide readers at MORDOR trial laboratories in Mangochi and Blantyre. Parasite density was estimated as parasites per microliter by assessing up to 100 high-power fields and assuming that 500 high-power fields contain the equivalent of one microliter of blood. If after two reads there was a discrepancy of greater than 20%, a third reading was taken as the final read. Thin slides were used to assess malaria species.

### Sample size.

Fifteen communities per arm, with 40 children sampled from each community (600 children per arm), provided 90% power to detect a reduction of malaria parasitemia from 20% to half that value at each study phase.

### Randomization and blinding.

Study drug was labeled with six letters by the manufacturer (Pfizer Inc., New York, NY), with three letters corresponding to azithromycin and three to placebo. Communities were randomly assigned to one of the six drug letters by the study statistician using the statistical package R (R Foundation for Statistical Computing, Vienna, Austria). All field and laboratory staff and participants in Malawi were blinded to the treatment code until after all data collection was complete.

### Statistical methods.

The main analysis was by intention-to-treat at the community level, in keeping with the cluster-randomized design of the MORDOR trial. Further analyses by both intention-to-treat and per-protocol at the individual level were used to further explore the data.

Community-level prevalence of parasitemia, anemia, and gametocytemia by treatment arm were assessed for the 12-month and 24-month visits using mixed-effects linear regression models including fixed effects for baseline prevalence and study phase and a random effect for community.

Individual-level parasitemia, parasite density, hemoglobin, and gametocytemia by treatment arm were assessed for the 12-month and 24-month visits using mixed-effects logistic or linear regression models, as appropriate, including fixed effects for age, baseline prevalence, and study phase and nested random effects for individuals within communities. Individual-level analyses were performed by both intention-to-treat and per-protocol, including only those who received study drug as indicated at the previous phase. Intra-class correlation coefficients (ICCs) were derived from the regression models at the level of the randomization unit.

The hemoglobin levels in individuals between the treatment arms were split by the presence of parasitemia and compared using Student’s *t*-test. Association between the presence of parasitemia and the hemoglobin level was assessed at the individual level using Student’s *t*-test.

### Ethical approval.

Ethical approval for morbidity assessments alongside the MORDOR trial was obtained from the College of Medicine, University of Malawi; the London School of Hygiene and Tropical Medicine; and the UCSF Committee on Human Research. Written consent was obtained from the guardians of participants. There were no incentives for participation.

## RESULTS

**Figure 1.**
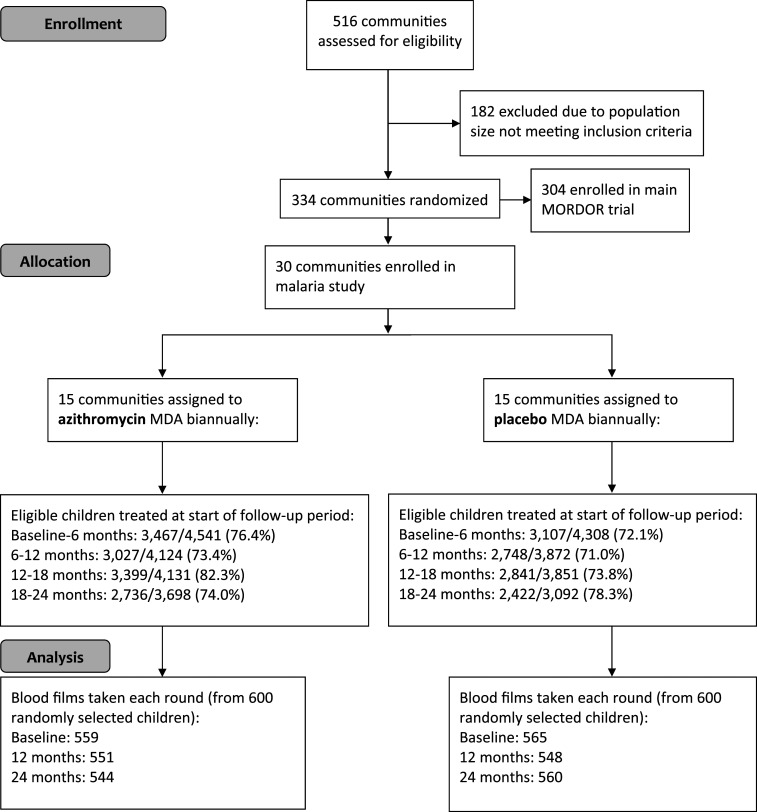
Trial flow. Communities were randomly selected from the same pool as the main MORDOR mortality study. Individuals could join the cohort at each of the biannual follow-up censuses.

Demographic details of sampled children in the 30 study communities are shown in Table 1. Age and gender distributions of sampled children were similar between the azithromycin- and placebo-treated communities. Study drug was distributed in all 30 communities at each round, with 76.6% of eligible children treated in azithromycin communities over all phases and 73.5% in placebo communities. The trial flow is shown in Figure 1, including the number of malaria blood films taken. No serious adverse events attributable to study drug were reported.

**Table 1:** Characteristics of children sampled in the study communities at the start of each follow-up period

	Placebo *N* (%)	Azithromycin *N* (%)
Age distribution (months)		
1–11	259 (47.9)	282 (52.1)
12–23	385 (51.2)	367 (48.8)
24–35	362 (50.2)	359 (49.8)
36–47	351 (51.1)	336 (48.9)
48–59	324 (50.2)	321 (49.8)
Gender		
Female	878 (50.3)	866 (49.7)
Male	841 (50.4)	828 (49.6)

Over 98% of malaria species detected were *Plasmodium falciparum*, and the remainder were *Plasmodium malariae*. Analyses included both malaria species. The community-level prevalence of malaria parasitemia, anemia, and gametocytemia was similar between placebo and azithromycin groups at baseline (unadjusted data shown in Table 2). Malaria parasitemia did not change significantly between treatment groups at the 12- and 24-month follow-up rounds: 1.0% lower in azithromycin-treated communities after adjusting for baseline parasitemia and follow-up phase (95% CI: −8.2 to 6.1%, *P* = 0.78; ICC = 0.51). The prevalence of anemia, defined as Hb < 11 g/dL, was not significantly different between treatment groups at the 12- and 24-month follow-up visits: 1.7% lower in azithromycin-treated communities (95% CI: −8.1 to 4.6%, *P* = 0.59; ICC = 0.09). The prevalence of gametocytemia also remained similar between treatment groups at the follow-up rounds: 0.7% lower in azithromycin-treated communities (95% CI: −2.8 to 1.5%, *P* = 0.53; ICC < 0.01).

**Table 2:** Community level prevalence of malaria parasitemia, anemia (Hb < 11 g/dL), and gametocytemia in the 30 study communities by treatment arm (unadjusted)

	Mean prevalence of parasitemia (95% CI)		Mean prevalence of anemia (95% CI)		Mean prevalence of gametocytemia (95% CI)	
Study phase	Placebo	Azithromycin	Placebo	Azithromycin	Placebo	Azithromycin
Baseline	29.2% (18.8–39.6%)	31.8% (21.8–41.8%)	58.1% (50.8–65.4%)	57.2% (50.1–64.2%)	6.2% (3.4–9.0%)	8.0% (4.3–11.7%)
12 months	34.8% (25.9–43.8%)	37.3% (27.7–47.0%)	59.2% (52.5–65.9%)	56.4% (49.7–63.2%)	5.0% (1.8–8.0%)	4.7% (2.3–7.1%)
24 months	29.2% (21.6–36.9%)	27.8% (18.6–37.0%)	51.7% (41.9–61.4%)	50.2% (43.5–56.8%)	3.5% (1.6–5.4%)	3.3% (0.6–6.1%)

Individual-level unadjusted data are shown by intention-to-treat in Table 3 and per-protocol in Table 4. The prevalence of parasitemia, parasite density, and hemoglobin was similar at baseline. By intention-to-treat, parasitemia was not significantly different between treatment groups at the 12- and 24-month follow-up visits: the odds ratio for individuals in azithromycin- compared with placebo-treated communities after adjusting for age, baseline parasitemia, and follow-up phase was 0.89 (95% CI: 0.53 to 1.50, *P* = 0.67; ICC = 0.06). Parasite density in parasitemic individuals was similar between treatment groups at the 12- and 24-month follow-up visits: 23 parasites/µL lower in individuals in azithromycin-treated communities after adjusting for age, baseline parasitemia, and follow-up phase (95% CI: −67 to 22 parasites/µL, *P* = 0.32; ICC = 0.02). Hemoglobin was also similar between treatment groups at 12- and 24-month follow-up visits: 0.08 g/dL lower in individuals in azithromycin-treated communities after adjusting for age, baseline community prevalence of anemia, and follow-up phase (95% CI: −0.36 to 0.19, *P* = 0.56; ICC = 0.05).

**Table 3:** Individual level parasitemia, parasite density in parasite-positive individuals, and hemoglobin analyzed by intention-to-treat (unadjusted)

	Prevalence of parasitemia				Parasite density (parasites/µL)				Hemoglobin (g/dL)			
	Placebo		Azithromycin		Placebo		Azithromycin		Placebo		Azithromycin	
Study phase	N*	Mean (95% CI)	N*	Mean (95% CI)	*N*	Mean (95% CI)	*N*	Mean (95% CI)	N*	Mean (95% CI)	N*	Mean (95% CI)
Baseline	565	28.5% (24.8–32.2%)	559	30.9% (27.1–34.8%)	161	352 (277–426)	173	368 (305–431)	564	10.5 (10.4–10.6)	559	10.6 (10.4–10.7)
12 months	548	34.5% (30.5–38.5%)	551	37.0% (33.0–41.1%)	189	195 (161–228)	204	172 (142–203)	547	10.5 (10.4–10.6)	549	10.5 (10.4–10.6)
24 months	560	29.3% (25.5–33.1%)	544	27.0% (23.3–30.8%)	164	214 (175–254)	147	200 (156–244)	558	10.8 (10.7–10.9)	544	10.8 (10.7–11.0)

* Different values between parasitemia and hemoglobin as a very small proportion of individuals did not have all tests.

**Table 4:** Individual level parasitemia, parasite density in parasite-positive individuals, and hemoglobin analyzed per-protocol, including only those who received treatment at the previous phase (unadjusted)

	Prevalence of parasitemia				Parasite density (parasites/µL)				Hemoglobin (g/dL)			
	Placebo		Azithromycin		Placebo		Azithromycin			Placebo		Azithromycin
Study phase	N*	Mean (95% CI)	N*	Mean (95% CI)	*N*	Mean (95% CI)	*N*	Mean (95% CI)	N*	Mean (95% CI)	N*	Mean (95% CI)
12 months	391	36.3% (31.5–41.1%)	404	36.4% (31.7–41.1%)	142	191 (153–228)	147	180 (141–220)	390	10.5 (10.3–10.7)	402	10.5 (10.4–10.7)
24 months	385	31.4% (26.8–36.1%)	367	23.7% (19.3–28.1%)	121	220 (171–268)	87	182 (139–224)	385	10.8 (10.7–11.0)	367	10.9 (10.8–11.1)

* Different values between parasitemia and hemoglobin as a very small proportion of individuals did not have all tests.

In the per-protocol analysis, the odds ratio for parasitemia in azithromycin- compared with placebo-treated individuals at 12- and 24-month visits after adjusting for age, baseline parasitemia, and follow-up phase was 0.71 (95% CI: 0.43 to 1.16, *P* = 0.17; ICC = 0.06). Parasite density in parasitemic individuals was similar between treatment groups at 12- and 24-month visits after adjusting for age, baseline parasitemia, and follow-up phase: 19 parasites/µL lower in azithromycin-treated individuals (95% CI: −45 to 8, *P* = 0.17; ICC = 0.03). Hemoglobin levels were also similar between treatment groups at the 12- and 24-month follow-up visits after adjusting for age, baseline community prevalence of anemia, and follow-up phase: 0.03 g/dL lower in azithromycin-treated individuals (95% CI: −0.32 to 0.25, *P* = 0.81; ICC = 0.04), shown in Table 4. There were no significant differences in gametocyte prevalence or gametocyte density in gametocyte-positive individuals when analyzed by intention-to-treat or per-protocol, shown in Supplemental Tables 1 and 2.

In the individual-level per-protocol and intention-to-treat analyses, there was a positive association between the outcomes (parasitemia and hemoglobin) and both age and baseline community parasitemia or hemoglobin. The effect of treatment did not vary with age when an interaction term between the treatment arm and age was included in the models.

Hemoglobin levels were approximately 1 g/dL lower in parasitemic individuals at all study phases and in azithromycin and placebo groups, shown in Supplemental Table 3.

## DISCUSSION

Several morbidity sub-studies were nested within the MORDOR trial to investigate mechanisms through which azithromycin MDA may reduce mortality. This research was not carried out in the MORDOR mortality study communities to reduce the risk of interventions impacting the primary MORDOR outcome of mortality. Morbidity study communities were randomly selected from the same pool as the main MORDOR trial, designed so that results would be representative of the mortality study. This malaria study, nested within MORDOR, did not identify a reduction in malaria parasitemia, gametocytemia, or hemoglobin in azithromycin-treated compared with placebo-treated communities. Secondary analyses at the individual level, by intention-to-treat and per-protocol, including only those who received study drug at the previous phase, approximately 6 months earlier, also did not identify significant differences in the prevalence of parasitemia or parasite density in parasitemic individuals.

Previous published reports of the effect of azithromycin MDA on malaria have suggested a reduction in malaria over 6-month duration.^4,5,7^ The mechanism through which a reduction in parasitemia over this time period may occur is unclear. Azithromycin displays delayed activity against the malaria parasite, preventing the progeny of antibiotic-treated parasites from fully maturing, and has a long terminal half-life of approximately 68 hours. These are properties that could feasibly contribute to a period of antimalarial activity of days to weeks but not months.^8^ The studies showing longer term effects were in Niger and the Gambia, in areas with lower background levels of parasitemia, which may be more amenable to reductions in transmission that could last longer than individual-level prophylaxis and treatment. This study assessed malaria gametocytemia as an effect of azithromycin on the sexual stages of the parasite which could explain the longer term community-level reductions in parasitemia as previously reported. Reductions in gametocytemia were not identified, which is consistent with previous evidence.^9^

In the context of the MORDOR trial showing a reduction in child mortality, this study does not provide evidence for an effect of azithromycin MDA on malaria mortality at the Malawi site.^1^ The best estimates for the odds ratio for the presence of parasitemia in individuals in azithromycin communities compared with placebo ones were 0.89 by intention-to-treat and 0.71 per-protocol, and it is possible that this study of limited size with follow-up restricted to 6 months post-MDA represents type II error. However, while the main MORDOR trial suggested increased child survival benefit in those younger than 6 months, in this study, age and parasitemia were positively associated (i.e., lower parasitemia in infants), further suggesting that the child survival benefits in MORDOR were not best explained through effects on malaria. Further investigation of the effects of azithromycin MDA on malaria severity and mortality, as well as cause-specific mortality for all the major causes of child mortality, is required to improve our understanding of whether the reported mortality reductions may be due to specific anti-pathogen effects or to other mechanisms, such as the immunomodulatory and anti-inflammatory effects of azithromycin.^10,11^

The prevalence of malaria parasitemia in this study was approximately 30% in children aged 1–59 months. Malawi is hyperendemic for malaria with 95% of the population susceptible to infection, and large-scale surveys before and after this study reported malaria parasitemia prevalence of 33% and 24%, respectively.^12,13^ Malaria was also the commonest inferred cause of death from verbal autopsy in the MORDOR trial in Malawi.^14^ Parasitemic children had significantly lower mean hemoglobin levels at all follow-up rounds, consistent with known effects of malaria as a cause of anemia in children.^15–17^ This analysis provides some validation of the integrity of the data. In addition, the ICCs were low for all analyses, suggesting considerable heterogeneity in malaria infection and hemoglobin levels by community, consistent with usual epidemiological patterns of malaria and similar to the MORDOR Niger site data.^7^

One limitation of this study is that treatment took place at the beginning and end of each dry season for logistical reasons. Mathematical modeling of the effect of azithromycin MDA on malaria in areas of highly seasonal transmission suggests there may be most benefit from treatment during the low-transmission season when treatment of established infection may produce more sustained benefit because of the lower risk of reinfection.^18^ At the community level, this may also reduce the reservoir of infectious individuals at the beginning of the following high-transmission period. In Malawi, where despite some seasonality, malaria prevalence remains high year-round, the optimal time to treat, if indeed there is any benefit from the intervention, is unclear.

Monitoring for infection took place during the baseline, 12-month, and 24-month visits, early in the dry season, and approximately 6 months after the previous treatment round for the latter two visits. This seasonality and timing of sample collection may not provide a complete understanding of the effect of the biannual azithromycin MDA on malaria prevalence, which may vary with time lapsed after administration. Finally, while carefully designed to be representative of the MORDOR trial site in Malawi, the data may not be representative of other countries with different malaria prevalence and healthcare interventions, although some data are available from other MORDOR sites.^7^ Any effect of azithromycin MDA on malaria may be impacted by other malaria interventions, such as seasonal malaria chemoprophylaxis (SMC), which was not taking place at the MORDOR-Malawi site. A recent study indicated no additional child mortality benefit when adding azithromycin MDA to SMC in Mali and Burkina Faso.^19^

In conclusion, a cluster-randomized placebo-controlled study of the effects of azithromycin MDA on malaria parasitemia was unable to detect a difference in malaria parasitemia at the community or individual levels, providing no evidence that any child survival benefit was mediated by effects on malaria mortality. Further investigation is required to understand the effect of azithromycin MDA on malaria morbidity and mortality and, indeed, to elucidate the mechanisms by which azithromycin MDA may reduce child mortality.

## Supplemental tables

Supplemental materials
