# Supplementary material for: Effects of Biannual Azithromycin Mass Drug Administration on Malaria in Malawian Children: A Cluster-Randomized Trial
Source: Am J Trop Med Hyg. 2020 Apr 27;103(3):1329–34. doi: 10.4269/ajtmh.19-0619 (PMC7470590; doi:10.4269/ajtmh.19-0619)
Supplement: Supplementary file 1 [file tpmd190619.SD1.docx]

**Supplemental Table 1:** Individual level gametocytemia and gametocyte density (gametocytes/µl) in gametocytemic children analysed by intention-to-treat (unadjusted)

|  | **Prevalence of gametocytemia** | | | | **Gametocyte density (gametocytes/µl)** | | | |
| --- | --- | --- | --- | --- | --- | --- | --- | --- |
|  | **Placebo** | | **Azithromycin** | | **Placebo** | | **Azithromycin** | |
| **Study phase** | **N** | **Mean (95%CI)** | **N** | **Mean (95%CI)** | **N** | **Mean (95%CI)** | **N** | **Mean (95%CI)** |
| Baseline | 565 | 6.0% (4.1-8.0%) | 559 | 8.1% (5.8-10.3%) | 34 | 18.0 (1.7-34.2) | 45 | 6.4 (3.7-9.1) |
| 12 months | 548 | 5.1% (3.3-7.0%) | 551 | 4.9% (3.1-6.7%) | 28 | 6.5 (4.0-8.9) | 27 | 5.8 (2.1-9.5) |
| 24 months | 559 | 3.4% (1.9-4.9%) | 544 | 3.3% (1.8-4.8%) | 19 | 12.6 (1.0-24.2) | 18 | 3.4 (1.6-5.1) |

Prevalence of gametocytemia: *P* = 0.35 comparing treatment arms at months 12 and 24 in mixed effects logistic regression model including fixed effects for age, mean baseline community prevalence of gametocytemia and study phase and nested random effects for individuals within communities [odds ratio in azithromycin compared to placebo-treated communities: 0.76 (95%CI 0.43 to 1.35); ICC = 0.04 (95% CI 0.01 to 0.20)]

Gametocyte density: *P* = 0.22 comparing treatment arms at months 12 and 24in mixed effects linear regression model including fixed effects for age, mean baseline community prevalence of gametocytemia and study phase and nested random effects for individuals within communities [3.3 gametocytes/µl lower in azithromycin-treated communities (95%CI -8.7 to 2.0); ICC < 0.01]

**Supplemental Table 2:** Individual level prevalence of gametocytemia and gametocyte density (gametocytes/µl) in gametocytemic children analysed per-protocol, including only those who received treatment at the previous phase (unadjusted)

|  | **Prevalence of gametocytemia** | | | | **Gametocyte density (gametocytes/µl)** | | | |
| --- | --- | --- | --- | --- | --- | --- | --- | --- |
|  | **Placebo** | | **Azithromycin** | | **Placebo** | | **Azithromycin** | |
| **Study phase** | **N** | **Mean (95%CI)** | **N** | **Mean (95%CI)** | **N** | **Mean (95%CI)** | **N** | **Mean (95%CI)** |
| 12 months | 391 | 5.1% (2.9-7.3%) | 404 | 5.9% (3.6-8.3%) | 20 | 6.4 (3.6-9.1) | 24 | 6.0 (1.8-10.2) |
| 24 months | 384 | 3.1% (1.4-4.9%) | 367 | 3.0% (1.2-4.7%) | 12 | 5.8 (2.5-9.0) | 11 | 2.8 (1.7-3.9) |

Prevalence of gametocytemia: *P* = 0.61 comparing treatment arms at months 12 and 24 in mixed effects logistic regression model including fixed effects for age, mean baseline community prevalence of gametocytemia and study phase and nested random effects for individuals within communities [odds ratio in azithromycin compared to placebo-treated communities: 0.86 (95%CI 0.47 to 1.54); ICC = 0.04 (95% CI <0.01 to 0.35)]

Gametocyte density: *P* = 0.50 comparing treatment arms at months 12 and 24 in mixed effects linear regression model including fixed effects for age, mean baseline community prevalence of gametocytemia and study phase and nested random effects for individuals within communities [1.2 gametocytes/µl lower in azithromycin-treated communities (95%CI -4.9 to 2.4); ICC 0.04]

**Supplemental Table 3:** Hemoglobin in parasite-positive and parasite-negative children

|  | **Placebo communities** | | | **Azithromycin communities** | | |
| --- | --- | --- | --- | --- | --- | --- |
| **Malaria parasitemia** | **N** | **Mean Hb (g/dl) (95%CI)** | ***P-*value*** | **N** | **Mean Hb (g/dl) (95%CI)** | ***P*- value*** |
| Baseline |  |  |  |  |  |  |
| Negative | 404 | 10.8 (10.7-11.0) | <0.0001 | 386 | 10.8 (10.7-11.0) | <0.0001 |
| Positive | 160 | 9.6 (9.4-9.9) |  | 173 | 10.0 (9.7-10.2) |  |
| 12 months |  |  |  |  |  |  |
| Negative | 359 | 10.8 (10.6-11.0) | <0.0001 | 345 | 10.8 (10.7-11.0) | <0.0001 |
| Positive | 188 | 9.9 (9.7-10.1) |  | 204 | 9.9 (9.7-10.2) |  |
| 24 months |  |  |  |  |  |  |
| Negative | 394 | 11.1 (11.0-11.3) | <0.0001 | 397 | 11.1 (11.0-11.2) | <0.0001 |
| Positive | 164 | 10.1 (9.9-10.3) |  | 147 | 10.1 (9.8-10.3) |  |

**P* values from Student’s t-test comparing mean Hb in malaria infected and non-infected individuals by study phase and treatment group.
